# Twist/untwist parameters are promising evaluators of myocardial mechanic changes in heart failure patients with preserved ejection fraction

**DOI:** 10.1002/clc.23353

**Published:** 2020-03-25

**Authors:** Yi Zhang, Shen‐Yi Li, Juan‐Juan Xie, Yuan Wu

**Affiliations:** ^1^ Department of Ultrasonography People's Hospital of Hunan Province Changsha China

**Keywords:** heart failure, left ventricular function, NT‐proBNP, speckle tracking echocardiography, twist

## Abstract

**Background:**

This study aimed to evaluate the twist/untwist parameters of the left ventricle (LV) in patients with heart failure with preserved ejection fraction (HFpEF) measured by ultrasonic two‐dimensional speckle tracking echocardiography (STE) and to examine the correlations between twist parameters and serum N‐terminal pro b‐type natriuretic peptide (NT‐proBNP) as well as conventional two‐dimensional echocardiography (2DE) indexes.

**Hypothesis:**

Changes in twist/untwist parameters can be used to evaluate LV function in HFpEF patients.

**Methods:**

In 63 HFpEF patients and 40 healthy controls, we analyzed LV twist/untwist parameters by STE, cardiac function by 2DE, and serum NT‐proBNP by enzyme‐linked immunosorbent assay (ELISA). The correlations between twist/untwist parameters and 2DE parameters and serum NT‐proBNP were examined by Pearson correlation analysis.

**Results:**

Left ventricular end diastolic inner diameter and ejection fraction in HFpEF patients were within the normal range, whereas other 2DE parameters including left ventricular posterior wall end diastolic thickness, interventricular septal thickness, left atrial volume index, E, E/A, and E/e' differed significantly between HFpEF patients and control subjects. The twist/untwist parameters such as peak apical rotation (Par), peak untwisting velocity (PUWV), and isovolumic diastole untwisting percentage (Iutw%) were significantly decreased in HFpEF patients compared with control participants. Positive correlations between PUWV/Iutw% and E/A/E/e' and a significant negative correlation between PUWV/Iutw% and left atrial volume index (LAVI) were observed. The plasma NT‐proBNP concentration was positively correlated with LAVI, but negatively correlated with PUWV and Iutw%.

**Conclusions:**

Changes in twist/untwist parameters correlate well with conventional 2DE parameters and plasma levels of NT‐proBNP, and can be used to evaluate LV function in HFpEF patients. Par is sensitive to the LV myocardial function damage.

## 1 INTRODUCTION

Heart failure with preserved ejection fraction (HFpEF) is characterized by clinical manifestations of heart failure while the left ventricular (LV) ejection fraction (EF) remains normal or only slightly impaired (EF ≥ 50%).^1^ Age‐related diseases such as hypertension and coronary heart disease are risk factors for HFpEF.^2^ The incidence of HFpEF is increasing with the aging population and attracting more clinical attention than before.^3^ Changes in the function and morphological structure of the heart are usually measured by conventional two‐dimensional echocardiography (2DE). Recently, speckle tracking echocardiography (STE) as a new technique has begun to be used more frequently in evaluating global and regional LV functions, and using STE has been shown to be more accurate for quantifying LV dysfunction than EF in HFpEF patients.^4^

Recent studies have employed STE to evaluate the LV long‐axis, short‐axis, and radial functions^5^, ^6^ as well as the left atrium (LA) functions,^7^, ^8^ but evaluation of LV twist/untwist motion in HFpEF patients has been rarely reported, and whether the twist/untwist motion parameters may be useful to evaluate cardiac function in HFpEF patients remains unknown. The major purpose of this study was to compare the LV twist parameters and conventional 2DE parameters as well as N‐terminal pro b‐type natriuretic peptide (NT‐proBNP) in assessing changes in cardiac function in HFpEF patients and to provide new approaches and viewpoints for clinical treatment and prognosis.

## 2 MATERIALS AND METHODS

### 2.1 Study population and design

A total of 63 patients who were diagnosed with HFpEF in the People's Hospital of Hunan province between 10 June 2015 and 10 June 2018 and 40 healthy controls were registered in our study. Diagnosis of HFpEF was based on the presence of symptoms and signs of heart failure and diastolic dysfunction without LV systolic dysfunction or dilatation. A “preserved” EF was defined as Left ventricular ejection fraction (LVEF) ≥50%. The definition of diastolic dysfunction included NT‐proBNP >220 pg/mL, the ratio of mitral valve early diastolic peak flow rate (E) to the tissue Doppler mean between the septal and lateral wall' (E/e′) >15, and/or other objective evidence of cardiac functional and structural alterations underlying Heart failure (HF), as defined by the 2015 consensus statement of the International Journal of Cardiology.^9^

The demographic and basal clinical characteristics of HFpEF patients and controls were obtained through retrieval from the hospital database or inquires. This study was approved by the ethics committee of our hospital, and all participants provided written informed consent.

### 2.2 Two‐dimensional echocardiography

2DE was performed using a GE Vivid E9 ultrasound system with a M5S transducer (GE Medical Healthcare, China), with a frame rate ≥50 frame/sec. The participants were in a calm state and in the left lateral position while 2DE was performed. The conventional 2DE parameters recorded included the left ventricular end diastolic inner diameter (LVDD), left ventricular posterior wall end diastolic thickness (LVPW), interventricular septal thickness (IVS), EF, left atrial volume index (LAVI), E, late diastolic peak flow rate (A), E/A, and E/e'.

### 2.3 Speckle tracking echocardiography

All participants were instructed to hold their breath at the end of exhalation, and short‐axis dynamic images of the LV apical and the basal plane of the heart were recorded for three consecutive cardiac cycles with a stable heart rhythm. Image acquisition was guided by the following internal landmarks: the apical plane was proximal to the underside of the papillary muscle at a level where it was not yet visible, and the basal plane was identified by the tip of mitral valve leaflets. The ventricular cavity was kept round as much as possible. The angular displacement of the LV in each short‐axis plane was defined as “LV rotation.” Apical rotation was defined as a positive value, and basal rotation was defined as a negative value. The net difference in LV rotation between the apical and basal plane was defined as “LV twist.” Thus, peak LV twist (Ptw) = peak apical rotation (Par) − peak basal rotation (Pbr). Untwisting, the directional reversal of systolic twist motion during diastole, and its angular displacement, are the same as twist, but the peak untwisting velocity (PUWV) is different from twist in systole. Most untwisting was completed during the isovolumic diastole period, so the percentage of untwisting in isovolumic relaxation (Iutw%) may reflect whether untwisting is delayed.

**Figure 1:**
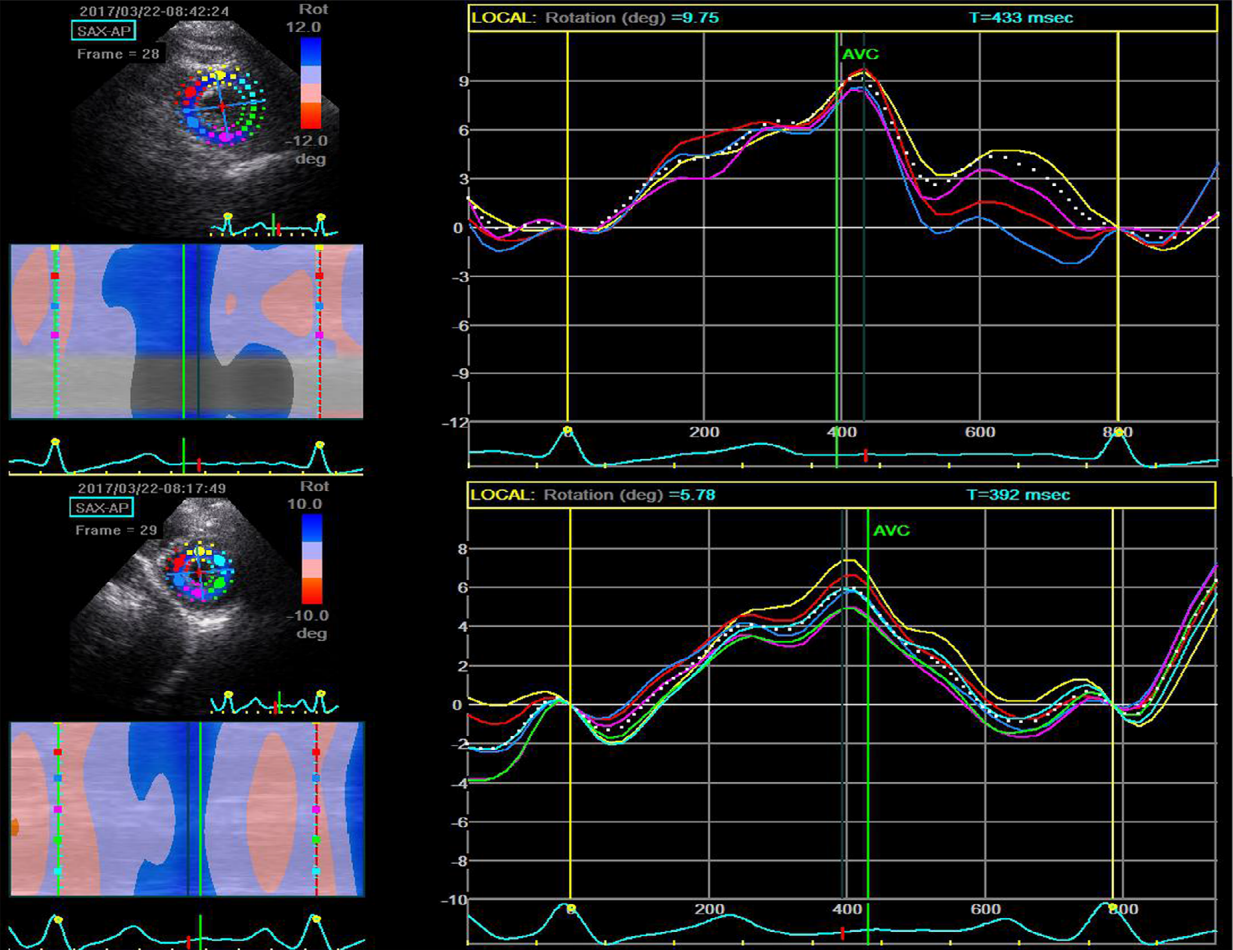
STE analysis showing peak apical rotation angle (Par) values of 9.75° for a normal subject and 5.78° for a heart failure with preserved ejection fraction patient

Twist parameters were analyzed offline by an experienced echocardiographer who blinded to clinical diagnosis and NT‐BNP, using the STE software (Echopac PC, Version, 6.0.2 GE Healthcare). The parameters included Par, basal rotation angle peak value (Pbr), twist angle peak value (Ptw), twist speed peak value (PtwV), and twist angle peak time as a percentage of systolic duration (TPK%). The measurement also included two diastole untwist parameters: PUWV and Iutw% (Figure 1).

### 2.4 Determination of serum NT‐proBNP

The plasma NT‐proBNP levels were measured by enzyme‐linked immunosorbent assay (ELISA) in all participants on the same day that 2DE was performed.

### 2.5 Statistical analysis

All statistical analyses were performed by the SPSS 13.0 software system (SPSS Inc., Chicago, Illinois). The data were expressed as mean ± SD, and the variance test was used to compare differences in data between the two groups. Pearson correlation analysis was performed to determine the relationships between twist/untwist parameters and conventional ultrasonic indexes as well as between twist/untwist parameters and the plasma NT‐proBNP concentration. A *P* value less than .05 was considered statistically significant.

## 3 RESULTS

### 3.1 Comparison of demographic and basal clinical characteristics between the HFpEF and control groups

A total of 63 HFpEF patients (20 males and 43 females) with an average age of 60.44 ± 8.43 years (range: 49‐74 years), and 40 controls (14 males and 26 females) with an average age of 58.05 ± 7.19 years (range: 45‐70 years), were enrolled in this study. Among the 63 patients, 37 had hypertension, 59 had coronary heart disease, and 17 had diabetes. No significant differences in the demographic and baseline clinical characteristics were observed between the two groups (Table 1, Supporting information).

### 3.2 Comparison of 2DE parameters between the HFpEF and control groups

The conventional 2DE parameters for participants in each group are shown in Table 1. The LVDD and EF did not differ between the HFpEF and control groups (*P* > .05), but the HFpEF group had significantly higher values of LVPW, IVS, LAVI, and E/e' as well as lower E and E/A values compared with the control group (*P* < .01); (Table 1, Supporting information), indicating that HFpEF patients did have cardiac dysfunction.

**Table 1:** Comparison of twist and untwist parameters between the control and HFpEF groups

	Control group (n=40)	HfpEF group (n=63)	F	P
Par (°)	9.20 ±1.61	8.46 ± 1.90	3.30	0.04
Pbr (°)	‐6.86 ± 1.90	‐6.47 ± 1.56	1.32	0.25
Ptw (°)	16.07 ± 2.85	14.93 ± 3.02	3.22	0.06
PtwV (°/s)	93.25 ± 11.59	89.40 ± 13.19	2.29	0.13
TPK% (%)	87.93 ± 6.61	85.27 ± 9.09	2.64	0.11
PUWV (°/s)	91.33 ± 14.57	76.20 ± 16.17	21.49	0.00
Iutw% (%)	55.14 ± 10.62	41.90 ± 14.12	38.59	0.00

Abbreviations: Par, apex rotation angle peak value; Pbr, bottom rotation angle peak value; Ptw, torsion angle peak value; PtwV, torsion speed peak value; TPK%, torsion angle peak time as a percentage of systolic duration; PUWV, untwisting speed peak value; Iutw%, isovolumic diastole untwisting percentage.

### 3.3 Comparison of twist/untwist parameters between the HFpEF and control groups

**Figure 2:**
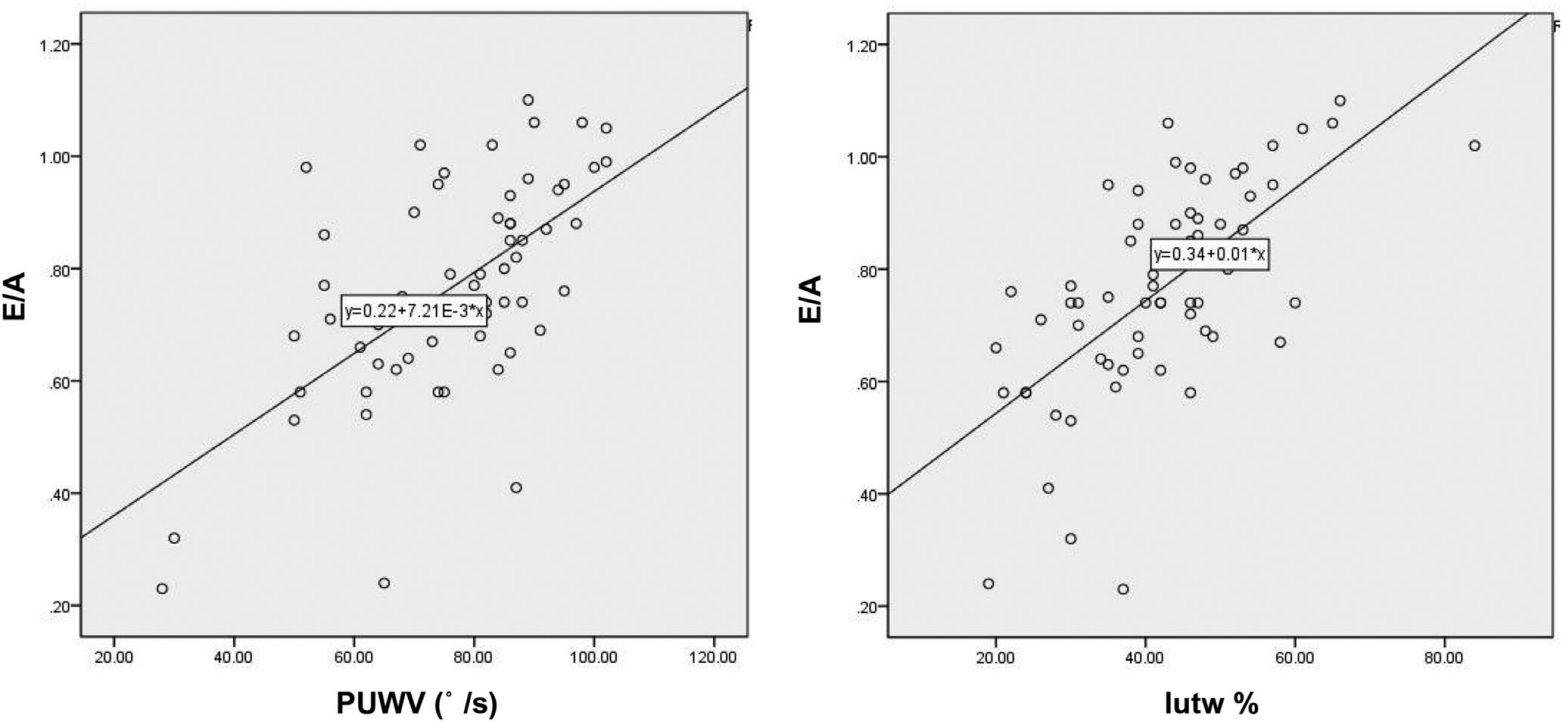
E/A exhibited positive correlations with peak untwisting velocity (PUWV) (left) and isovolumic diastole untwisting percentage (Iutw%) (right)

Next, STE was performed on each participant. While some systolic parameters such as basal rotation angle peak value (Pbr), Ptw, PtwV, and TPK% were comparable between the HFpEF and control groups (*P* > .05), Par was significantly decreased during the contraction period in the HFpEF group compared with that in the control group (*P* < .01). Also, the diastolic untwist parameters PUWV and Iutw% in HFpEF patients were decreased significantly compared with those in control subjects (*P* < .01) (Table 1, Figures 1 and 2, Supporting information), suggesting that HFpEF patients had impaired cardiac diastolic function.

### 3.4 Determination of correlation between twist parameters and 2DE parameters

**Figure 3:**
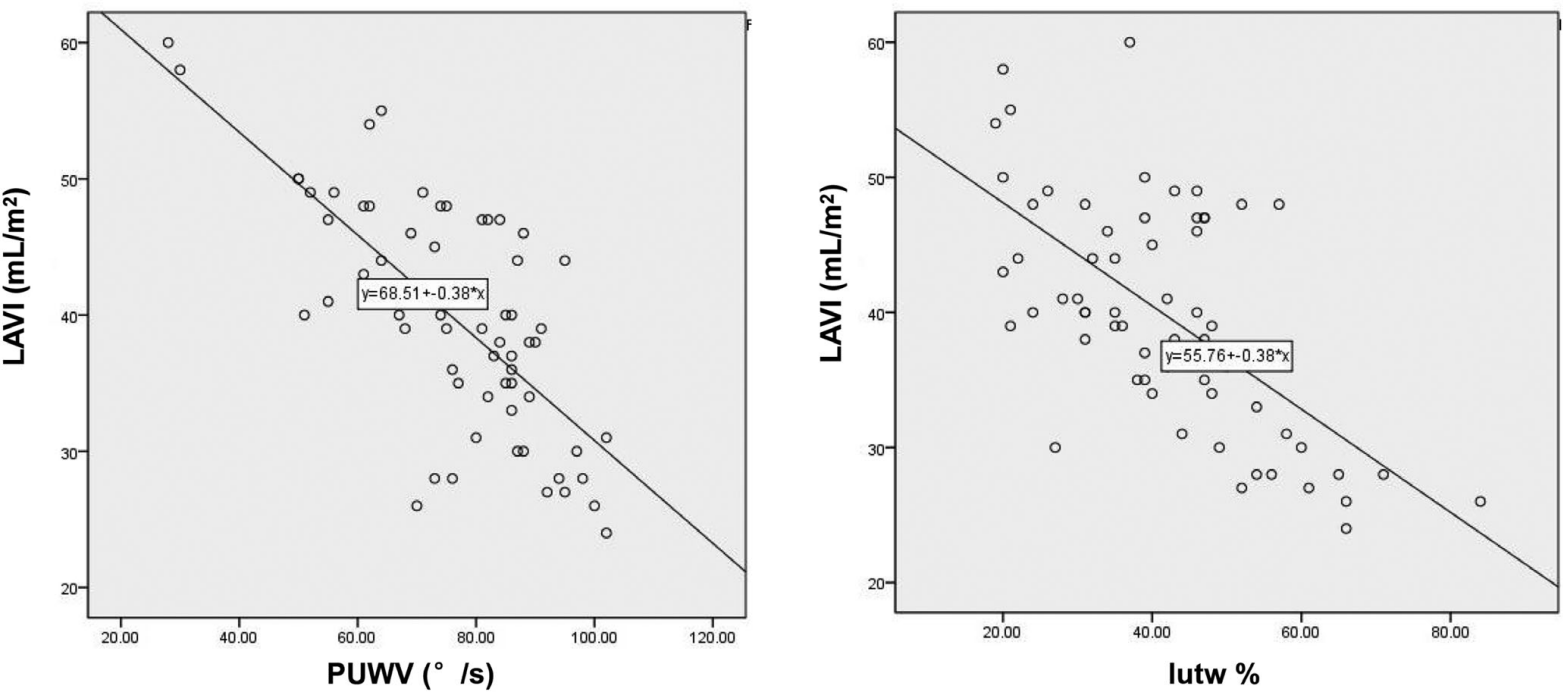
Left atrial volume index exhibited a positive correlation with peak untwisting velocity (PUWV) (left) but a negative correlation with isovolumic diastole untwisting percentage (Iutw%) (right)

We next used linear correlation analysis to examine the correlations between twist parameters and 2DE parameters. EF exhibited a certain level of correlation with Ptw (correlation coefficient, 0.47) but not with Pbr, PtwV, or TPK. The diastolic untwist parameters PUWV and Iutw% were positively correlated with E/A (correlation coefficients of 0.61 and 0.65, respectively) (Figure 2). Also, a negative correlation was found between the untwist parameters PUWV and Iutw% and LAVI (correlation coefficients of −0.72 and −0.64, respectively) (Figure 3).

### 3.5 Determination of correlation between NT‐proBNP and 2DE/twist parameters

**Figure 4:**
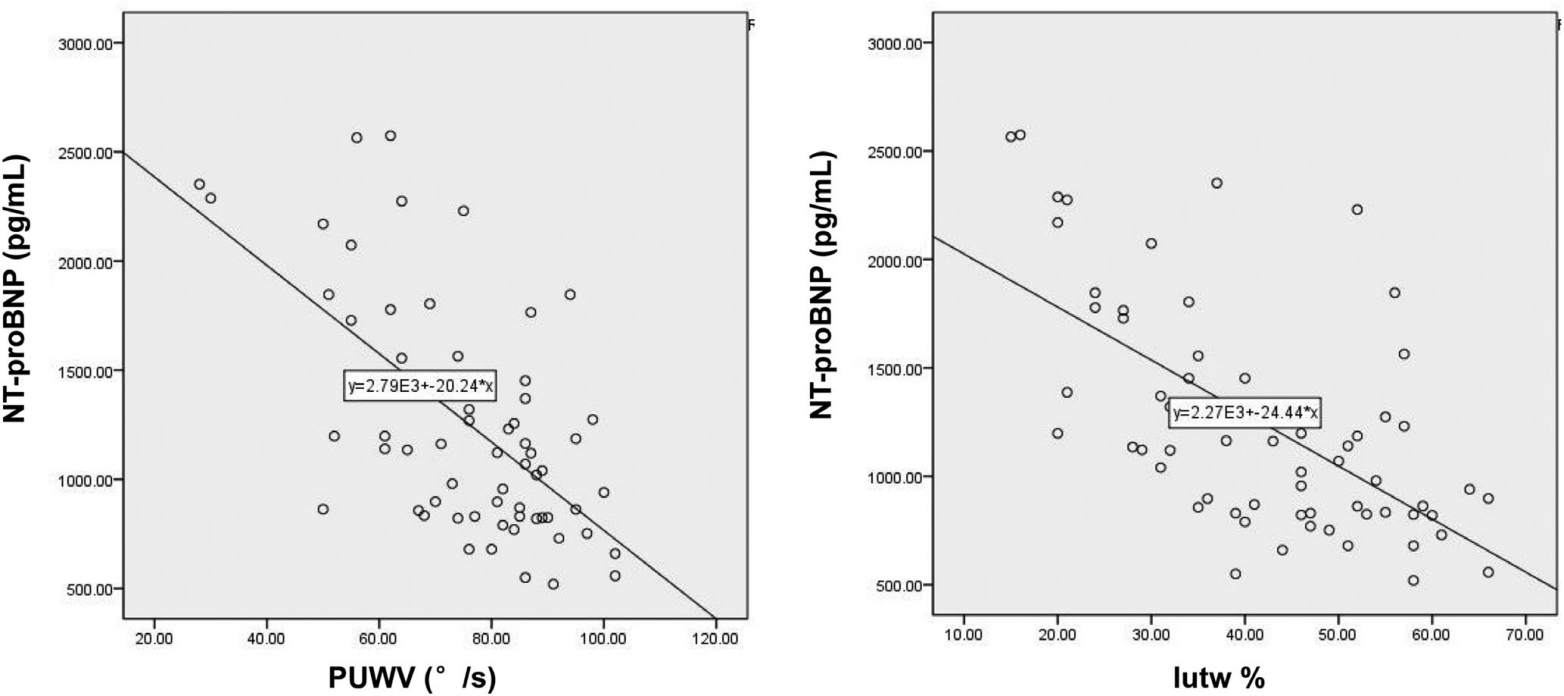
N‐terminal pro b‐type natriuretic peptide exhibited negative correlations with peak untwisting velocity (PUWV) (left) and Iutw% (right)

As expected, the plasma NT‐proBNP concentration in the HFpEF group was significantly greater than that in the control group (1247.24 ± 530.38 pg/mL vs 50.24 ± 15.86 pg/mL, *P* < .05). Correlation analysis showed that the plasma NT‐proBNP concentration was positively correlated with the LAVI (correlation coefficient, 0.64), but negatively correlated with PUWV and Iutw% (correlation coefficients of −0.62 and −0.63, respectively) (Figure 4).

## 4 DISCUSSION

Although the pathophysiologic mechanisms underlying HFpEF remain unclear, HFpEF patients are more likely to have LV hypertrophy or concentric remodeling, LV diastolic dysfunction, and enlargement of the LA.^10^ It is recognized that the main pathophysiological changes of HFpEF are the LV damage‐induced impairment of relaxation and calcium overload‐induced increase in myocardial stiffness, leading to cardiac diastolic dysfunction, prolongation of LV isovolumic diastole, slow filling, an increase in ejection resistance, and a rise in end diastole pressure.^11^ In the present study, we examined the correlations of twist/untwist parameters obtained by STE with 2DE parameters and serum NT‐proBNP and found that: (a) the twist/untwist parameters Par, PUWV, and Iutw% were significantly decreased in HFpEF patients compared with healthy controls; (b) PUWV and Iutw% were positively correlated with E/A and E/e′ but negatively correlated with LAVI; and (c) plasma NT‐proBNP was positively correlated with LAVI but negatively correlated with PUWV and Iutw%. Thus, we believe that twist/untwist parameters may be used to evaluate the severity of LV diastolic dysfunction in HFpEF patients with a new viewpoint.

Conventional 2DE has become a routine technique for assessing LV diastolic dysfunction and holds advantages such as noninvasiveness, simple operation, and accuracy. The conventional 2DE parameters most commonly assessed include E/A, E/e', and LAVI. The change in E/A reflects the degree of impairment of LV diastolic function, but the value of this index is easily affected by other factors such as heart rate, myocardial systolic and diastolic state, and valve regurgitation. E/e' is a valuable evaluation index for LV diastolic dysfunction and is not affected by changes in front and post load, heart rate, and other hemodynamic factors.^12^ In the present study, we observed alteration in a number of conventional 2DE parameters in the HFpEF group, and these observations were in line with a previous report and further confirmed the accuracy of the diagnosis of the patients enrolled in this study.^13^

The main pathological change in HFpEF is cardiac diastolic dysfunction, that is, LV stiffness increases, while the LA expands simultaneously, thus impairing the cardiac diastolic function. The main manifestation of LA dysfunction is the increase in its volume, which is an indicator of the severity of HFpEF and plays an important role in disease progression in HFpEF patients.^14^, ^15^ The LAVI is the volume of the LA corrected by body surface area; it eliminates interindividual differences in height and body mass. Thus, the LAVI accurately reflects the degree of LV diastolic dysfunction.^16^ In the present study, we observed that decreases in the diastolic untwist parameters PUWV and Iutw% were negatively correlated with the LAVI in HFpEF patients. Therefore, PUWV and Iutw% can also be used as indicators to assess the degree of LV diastolic dysfunction.

LV twist is defined as the wringing motion whereby the LV apex rotates with respect to the LV base around the LV long axis.^17^ Twist motion is an important component of myocardium movement, and recently it has often been used to evaluate early myocardial dysfunction both globally and locally.^18^ In HFpEF patients, LV local systolic function is damaged, but the global LV ejection function is preserved, and STE can be used to measure changes in rotation parameters, thus providing new evidence for a dysfunctional LV.^19^ Previous studies have shown that HFpEF patients had impaired systolic as well as diastolic function, and that LV systolic longitudinal functional reserve was significantly lower in this disease, indicating that HFpEF is not an isolated disorder of diastole.^20^ Our study showed that Par measured during the systolic period was decreased significantly in HFpEF patients compared with the control group, indicating that Par was sensitive to changes related to myocardial damage, and STE measurements of twist/untwist motion may have better sensitivity for detecting subtle functional deficits in LV contraction after myocardial injury when compared with conventional 2DE parameters.

The LV stores force in systole and releases it during the isovolumic relaxation by recoiling and taking blood rapidly into the LV during mitral valve opening. Untwist movement in the vast majority of healthy people is completed during the isovolumic diastole period and is related to the rapid release of untwist force into the extracellular matrix and the restoration of the original length of the shortened sarcomere.^21^ Hence, untwist movement determines the myocardial relaxation during the isovolumic diastole period and promotes blood flow from the LA to the LV. In HFpEF patients, the transport of calcium ions into myocardial cells during the diastolic period is altered, and the return of myocardial cells to their original length is compromised, both of which contribute to the slowdown of the untwist speed.^22^ Furthermore, the untwist movement is delayed, with most of them being completed after the isovolumic diastole period, and thus, the percentage of completed untwist during the isovolumic diastole period (Iutw%) is reduced.^22^ Tan et al show that in HFpEF, exercise limitation is due to combined systolic and diastolic abnormalities, particularly involving ventricular twist and deformation (strain), which mainly cause reduced ventricular suction, delayed untwisting, and impaired early diastolic filling.^20^ Consistent with the above findings, in the present study, we also found that HFpEF patients had significantly decreased PUWV and Iutw% compared with the control group, and these factors showed a negative correlation with LAVI but a positive correlation with E/A. Hence, the diastolic unwinding in HFpEF patients was related to the LV functionality. These findings suggest that diastolic parameters are sensitive to LV dysfunction in HFpEF patients.

NT‐proBNP is the amino acid residue of brain sodium urea precursor and is mainly secreted by the heart; especially the LV plasma NT‐proBNP levels may be used to help with diagnosing and evaluating the severity of cardiac diseases and holds a prognostic value for the outcomes of HF patients. An elevated plasma NT‐proBNP concentration suggests a cardiac hemodynamic disorder.^23^ Previous studies have shown that NT‐proBNP is an indicator of excess cardiac volume, and its elevation suggests diastolic dysfunction. Moreover, an increased circulating NT‐proBNP level in HFpEF patients is directly related to the increased LV diastolic filling pressure and end diastolic wall stress.^24^, ^25^ In addition, a significant correlation between the plasma NT‐proBNP concentration and E/e' in patients with HFpEF has been reported, and both parameters can accurately reflect LV diastolic dysfunction.^26^ Therefore, clinicians can determine the severity of cardiac fibrosis and actual dysfunction in HFpEF patients via combined evaluation of the serum NT‐proBNP level and clinical manifestations.^27^ In the present study, we found that the plasma NT‐proBNP concentration was negatively correlated with PUWV and Iutw%, indicating that the untwist parameters PUWV and Iutw% can be used to appraise LV diastolic dysfunction in HFpEF patients with a new prospective.

### 4.1 Study Limitations

In the present study, we did not take into account the patients' medication history, and this might may affect the results to some extent, because some medications might have influenced the LV systolic and diastolic functions. Also, our study had a limited sample size, which might have contributed to the finding of nonsignificant differences in the rotation angle, twist angle, and velocity and peak time percentage of the bottom of the heart between the groups.

## 5 CONCLUSION

Twist/untwist parameters may be used to assess the severity of LV diastolic dysfunction in HFpEF patients in a new viewpoint, although conventional 2DE shows that their EF is in the normal range. The systolic apical rotation angle is very sensitive to changes in LV myocardial mechanics and can be used as an early diagnostic indicator of myocardial dysfunction in HFpEF patients.

## DATA AVAILABILITY

The data that support the findings of this study are available from the corresponding author upon reasonable request.

## CONFLICT OF INTEREST

The authors declare no potential conflict of interest.

## Supporting information


**Supplementary Figure 1**The diastolic untwist parameter PUWV in the HFpEF group is significantly lower than that in the control group.

**Supplementary Figure 2**The diastolic untwist parameter Iutw% in the HFpEF group is significantly lower than that in the control group.

**Supplementary Table 1**Comparison of demographic and basal clinical characteristics as well as conventional ECG parameters between the control and HFpEF groups

**Supplementary Table 2**Comparison of twist and untwist parameters between the control and HFpEF group
