# Supplementary figures and images for: Twist/untwist parameters are promising evaluators of myocardial mechanic changes in heart failure patients with preserved ejection fraction
Source: Clin Cardiol. 2020 Mar 25;43(6):587–93. doi: 10.1002/clc.23353 (PMC7298990; doi:10.1002/clc.23353)

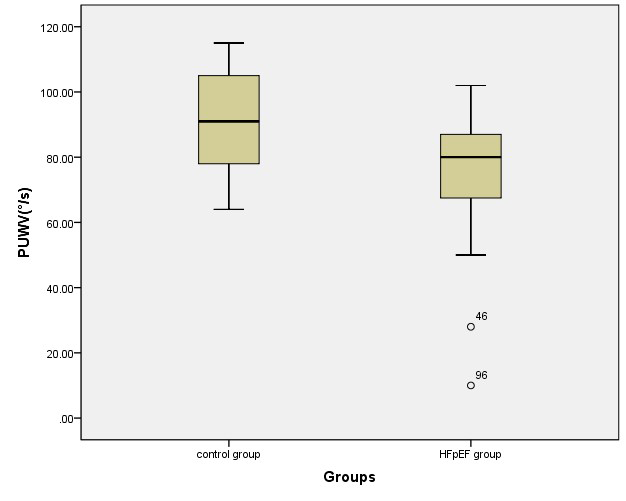

Supplement: Supplementary file 1 — Supplementary Figure 1The diastolic untwist parameter PUWV in the HFpEF group is significantly lower than that in the control group. [file CLC-43-587-s001.tif]

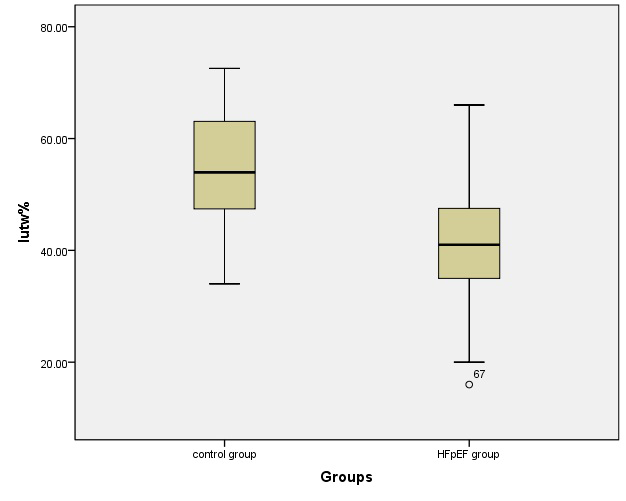

Supplement: Supplementary file 2 — Supplementary Figure 2The diastolic untwist parameter Iutw% in the HFpEF group is significantly lower than that in the control group. [file CLC-43-587-s002.tif]
