# Supplementary material for: Twist/untwist parameters are promising evaluators of myocardial mechanic changes in heart failure patients with preserved ejection fraction
Source: Clin Cardiol. 2020 Mar 25;43(6):587–93. doi: 10.1002/clc.23353 (PMC7298990; doi:10.1002/clc.23353)
Supplement: Supplementary file 3 — Supplementary Table 1Comparison of demographic and basal clinical characteristics as well as conventional ECG parameters between the control and HFpEF groups [file CLC-43-587-s003.doc]

**Supplementary Table 1.** Comparison of demographic and basal clinical characteristics as well as conventional 2DE parameters between the control and HFpEF groups

|  | Control group  (n=40) | HFpEF group  (n=63) | *F* | *P* |
| --- | --- | --- | --- | --- |
| Age, years (mean ± SD) | 58.05 ± 7.19 | 60.44 ± 8.43 | 1.545 | 0.217 |
| Height (cm) | 160.15 ± 7.45 | 161.11 ± 9.40 | 0.30 | 0.59 |
| Weight (kg) | 61.63 ± 7.28 | 64.54 ± 8.16 | 3.39 | 0.07 |
| HR (bpm) | 74.43 ± 8.32 | 71.62 ± 9.95 | 2.20 | 0.14 |
| LVDD (mm) | 44.40 ± 3.56 | 45.27 ± 3.40 | 1.54 | 0.22 |
| LVPW (mm) | 9.87 ± 0.79 | 11.02 ± 1.11 | 32.3 | 0.00 |
| IVS (mm) | 10.09 ± 0.83 | 11.28 ± 1.08 | 35.39 | 0.000 |
| EF (%） | 64.23 ± 5.53 | 61.81 ± 7.83 | 2.89 | 0.09 |
| LAVI (ml/m^2^) | 26.08 ± 3.48 | 39.76 ± 8.50 | 110.65 | 0.00 |
| E (cm/s) | 77.20 ± 13.35 | 60.63 ± 10.15 | 50.87 | 0.00 |
| E/A | 1.33 ± 0.14 | 0.76 ± 0.19 | 345.88 | 0.00 |
| E/eʹ | 6.38 ± 0.81 | 9.56 ± 2.20 | 76.58 | 0.00 |

Abbreviations: LVDD, left ventricular end diastolic inner diameter; LVPW, left ventricular posterior wall end diastolic thickness; IVS, ventricular septal thickness; EF, ejection fraction; LAVI, left atrial volume index; E, mitral valve early diastolic peak flow rate; A, late diastolic peak flow rate; E/e', ratio of E to Tissue Doppler mitral annulus early diastolic peak velocity.
