# Supplementary material for: Twist/untwist parameters are promising evaluators of myocardial mechanic changes in heart failure patients with preserved ejection fraction
Source: Clin Cardiol. 2020 Mar 25;43(6):587–93. doi: 10.1002/clc.23353 (PMC7298990; doi:10.1002/clc.23353)
Supplement: Supplementary file 4 — Supplementary Table 2Comparison of twist and untwist parameters between the control and HFpEF group [file CLC-43-587-s004.doc]

**Supplementary Table 2.** Comparison of twist and untwist parameters between the control and HFpEF group

|  | Control group  (n=40) | HFpEF group  (n=63) | *F* | *P* |
| --- | --- | --- | --- | --- |
| Par (°) | 9.20 ±1.61 | 8.46 ± 1.90 | 3.30 | 0.04 |
| Pbr (°) | -6.86 ± 1.90 | -6.47 ± 1.56 | 1.32 | 0.25 |
| Ptw (°) | 16.07 ± 2.85 | 14.93 ± 3.02 | 3.22 | 0.06 |
| PtwV (°/s) | 93.25 ± 11.59 | 89.40 ± 13.19 | 2.29 | 0.13 |
| TPK% (%) | 87.93 ± 6.61 | 85.27 ± 9.09 | 2.64 | 0.11 |
| PUWV (/s) | 91.33 ± 14.57 | 76.20 ± 16.17 | 21.49 | 0.00 |
| Iutw% (%) | 55.14 ± 10.62 | 41.90 ± 14.12 | 38.59 | 0.00 |

Abbreviations: Par, apex rotation angle peak value; Pbr, bottom rotation angle peak value; Ptw, torsion angle peak value; PtwV, torsion speed peak value; TPK%, torsion angle peak time as a percentage of systolic duration; PUWV, untwisting speed peak value; Iutw%, isovolumic diastole untwisting percentage.
